# Establishment and characterization of a primary cell culture derived from external auditory canal squamous cell carcinoma

**DOI:** 10.1002/2211-5463.13225

**Published:** 2021-06-29

**Authors:** Yuki Sekino, Akira Imaizumi, Noritaka Komune, Mayumi Ono, Kuniaki Sato, Shogo Masuda, Akiko Fujimura, Kensuke Koike, Takahiro Hongo, Ryutaro Uchi, Hideya Onishi, Takashi Nakagawa

**Affiliations:** ^1^ Department of Otorhinolaryngology Graduate School of Medical Sciences Kyushu University Fukuoka Japan; ^2^ Department of Cancer Therapy and Research Graduate School of Medical Sciences Kyushu University Fukuoka Japan

**Keywords:** external auditory canal, primary cell culture, squamous cell carcinoma, stem cell, temporal bone

## Abstract

There are no human cancer cell lines of external auditory canal origin available for research use. This report describes the establishment of a culture condition for external auditory canal squamous cell carcinoma, derived from human tumor tissue. Successive squamous cell carcinoma colonies were dissociated by trypsin, subcultured, and maintained on a feeder layer (MMC‐TIG‐1‐20), yielding a clonally proliferating cell culture. Two morphological types of colony were observed: (a) densely packed colonies and (b) colonies with indistinct boundaries characterized by cell–cell complexes with fibroblast feeder cells. The SCC‐like characteristics of these cells were evidenced by positivity for p53, SCCA1/2, cytokeratin, and vimentin, and cancer stem cell properties were indicated by positivity for CD44, CD133, Oct3/4, and alkaline phosphatase (ALP). One of the unique properties of cell cultures is their tendency to form steric colonies *in vitro* on feeder layer cells. In addition, in the presence of fresh macrophages, the cells very slowly transform to break away from colonies as free cells, a process that resembles the epidermal–mesenchymal transition, whereby cell–cell interactions are weakened and migration activity is enhanced. These factors are purported to play a key role in cancer cell metastasis.

## Graphical abstract

No human cancer cell lines of external auditory canal (EAC) origin are available for research use. This report describes the establishment of a culture condition for EAC squamous cell carcinoma (SCC). These cell cultures had cancer stem cell properties and tended to form steric colonies on feeder cells. These cell cultures could be used in future research on EAC‐SCC.

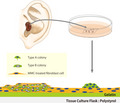

**Abbreviations**
EAC: external auditory canal
FSP: fibroblast surface protein
PBMC: Peripheral blood mononuclear cell
PDC: poorly differentiated cluster
SCC: squamous cell carcinoma
STR: short tandem repeat
VAF: variant allele frequency

Squamous cell carcinoma (SCC) of the external auditory canal (EAC‐SCC) is one of the rarest head and neck SCCs [1], with an estimated incidence of only 1 in 1 million [2]. The prognosis of advanced‐stage EAC‐SCC, in particular, remains poor, despite near‐daily progress in diagnostic imaging and treatment strategies for head and neck cancers.

Cell lines, which are used for the elucidation of carcinogenic mechanisms and efficacy tests for therapeutic drugs [3], are a very powerful tool in cancer research. Several cell lines have helped clinicians to collect evidence for the diagnosis and treatment of head and neck cancers (*e.g*., Hep‐2 [laryngeal carcinoma] [4], Ca9‐22 [oral carcinoma] [5], and SAS [tongue carcinoma] [6]). However, no such cell line has been established for EAC‐SCC. The establishment of an EAC‐SCC cell line is an essential first step in the construction of a human chimeric mouse model. This is one reason why *in vivo* and *in vitro* research into the disease’s treatment has lagged behind other head and neck cancers, and why its outcomes have failed to improve.

Bryne *et al*. report, on their studies of squamous cell carcinoma of the head and neck, that the invasive ability, not the degree of differentiation of cancer cells, is an important factor for the assessment of clinical aggressiveness and prognosis [7, 8]. Grade or Score 4 infiltration patterns show a widespread cellular dissociation in small groups and/or in single cells. The concept of tumor budding, which is characterized by the presence of a single cell or tumor cluster of tumor cells (< 5 cells) at the invasive front, has been introduced as an evaluation standard of head and neck SCC [9]. Tumor budding has been evaluated only for tumor masses of < 5 cells, but there are clusters of 5 or more cancer cells at the invasive front. Poorly differentiated clusters (PDCs) are tumor stroma that are histologically defined as tumor cell clusters composed of five or more tumor cells and having no gland‐like structure. This PDC is a negative prognostic factor established in several types of cancer [10, 11, 12]. In addition, a high incidence of PDC is associated with poor prognosis and recurrence of liver, lung, lymph node/local recurrence, and peritoneal dissemination [10].

In the present study, we succeeded in culturing human‐derived EAC‐SCC cells. The established cell cultures harbored cells with two types of morphologies. Unlike normal SCC cells, our cell cultures form amorphous cell clusters only on feeder cells, not on the culture vessel surface. When the cell size reaches a certain level, some cells are dispersed around the host cluster and proliferate by creating satellite clusters. We hypothesize that cells with this morphology possess the characteristic properties of cancer stem cells, because they are derived from a deep tumor in a hypoxic environment [13] and express cancer stem cell markers [14, 15, 16, 17].

## Materials and methods

### Ethic statement

The Clinical Research Ethics Review Committee of Kyushu University Hospital approved the study (permit no. 30‐268). Written informed consent for the current research project was obtained before harvesting the tumor tissue and blood sample. This study was also conducted according to the principles of the Declaration of Helsinki.

### Patients

Tumor tissue was collected for histological diagnoses. One patient (case 1) was a 69‐year‐old woman with well‐differentiated SCC of the left external auditory canal (clinical stage: cT3N1M0). The other patient (case 2) was a 71‐year‐old woman with well‐to‐moderately differentiated SCC of the right external auditory canal (clinical stage: cT4N0M0). These samples were used to create the cell cultures.

### Growth medium

The growth medium consisted of Dulbecco’s modified Eagle’s medium (DMEM)/F12 (Sigma‐Aldrich, St. Louis, MO, USA), supplemented with 10% fetal bovine serum (FBS: Sigma‐Aldrich), 0.1 mm MEM nonessential amino acids solution (Thermo Fisher Scientific, Waltham, MA), 1 mm sodium pyruvate (Thermo Fisher Scientific), 2 mm L‐glutamine (Thermo Fisher Scientific), and 1% antibiotic‐antimycotic mixed stock solution (Nacalai Tesque, Inc., Kyoto, Japan).

### Antibodies

Tables 1 and 2 shows the antibodies used for the cultured cells (Table 1) and paraffin‐embedded specimens (Table 2).

**Table 1:** Antibodies for immunocytochemistry.

Antibody	Clone	Host	Dilution for ICC	Source
Primary antibodies				
pan‐Cytokeratin‐Alexa 488	C11	Mouse	1 : 50	Santa Cruz Biotechnology, sc‐8018 AF488
Vimentin‐PE	V9	Mouse	1 : 50	Santa Cruz Biotechnology, sc‐6260 PE
p53‐Alexa 488	7F5	Rabbit	1 : 50	Cell Signaling Biotechnology, #5429
CD44‐PE	IM7	Rat	1 : 50	Tonbo biosciences, 50‐0441
CD133‐APC	AC133	Mouse	1 : 50	Miltenyi Biotec, 130‐113‐106
SCCA1/2	4C8	Mouse	1 : 50	LifeSpan BioSciences, LS‐C172653
Oct‐3/4	C‐10	Mouse	1 : 50	Santa Cruz Biotechnology, sc‐5279
Secondary antibody				
Alexa Fluor^®^ 555 anti‐mouse IgG		Goat	1 : 1000	Invitrogen, A21424

ICC, Immunocytochemistry.

**Table 2:** Antibodies for immunohistochemistry.

Antibody	Clone	Host	Dilution for IHC	Source
Primary antibodies				
p53(IgG)	DO‐7	Mouse	1 : 100	Dako, M7001
SCCA1/2(IgG)	4C8	Mouse	1 : 150	LifeSpan BioSciences, LS‐C172653
CD44(IgG)	2C5	Mouse	15 µg·mL^−1^	R&D Systems, BBA10
Human fibroblast surface protein (IgM)	1B10	Mouse	1 : 100	Sigma‐Aldrich, F4771
Secondary antibodies				
Biotinylated anti‐mouse IgG		Horse	1 : 1000	Vector Laboratories, BA‐2001
Biotinylated anti‐mouse IgM		Goat	1 : 1000	Vector Laboratories, BA‐2020

IHC, Immunohistochemistry.

### Cell lines

HeLa cells were obtained from the RIKEN BRC Cell Bank for use as the cytokeratin‐positive control in the immunostaining experiments; SCC‐9 cells were obtained from ATCC for use as the p53 and SCCA1/2‐positive control. Both lines were maintained in the growth medium at 37 °C with 5% CO_2_.

### Primary human fibroblasts

Primary human fibroblasts were prepared from the primary culture of SCC with a 200 µL micropipette tip under a microscope. Collected cells were then cultured for use as the CD44‐ and vimentin‐positive control and as the p53‐, SCCA1/2‐, CD133‐, Oct3/4‐negative control in the immunostaining experiments.

### Feeder culture (MMC‐treated TIG‐1‐20)

TIG‐1‐20 cells were obtained from the JCRB Cell Bank (National Institutes of Biomedical Innovation, Health and Nutrition). When the cells reached 80% confluence, they were treated with 10 µg·mL^−1^ of mitomycin C (MMC: FUJIFILM Wako Pure Chemical Corporation, Osaka, Japan) [18]. After 2.5‐h incubation, they were washed, disassociated by trypsin digestion, and seeded on a 60‐mmΦ dish (1.5 × 10^5^ cells per dish).

### Fresh human macrophages

Peripheral blood was obtained from a consenting healthy donor. Peripheral blood mononuclear cells (PBMCs) were isolated from whole blood by density centrifugation and seeded onto EAC‐SCC cell cultures at 1 × 10^6^ cells·mL^−1^ (3 mL per 60 mmΦ dish). The EAC‐SCC cell cultures were maintained by changing the medium every 3 days after the addition of PBMCs (lymphocytes, monocytes, macrophages, and dendritic cells). Free‐cell components (lymphocytes and some dendritic cells) were removed by refreshing the culture medium. In addition, since T lymphocytes die very quickly in the absence of stimulation [19], macrophages and dendritic cells soon became the only PBMC‐derived cells in this mixed culture.

### Immunofluorescence and alkaline phosphatase staining

Cell samples for immunostaining were prepared as follows. Single colonies from cell cultures were collected under a microscope and dispersed with trypsin. From this cell suspension, a single‐layer specimen of cells was prepared on a glass slide using Cytospin 4^®^ (Thermo Fisher Scientific). A slide for the control cell samples, which included SCC9 cells and human fibroblasts for p53, SCC1/2, CD44, CD133, and Oct3/4 staining, were prepared with cytospin according to the protocol used for EAC‐SCC cells. Immediately after centrifugation, cells and fragments were fixed with methanol : acetone 1 : 1 (v/v) for 20 min at −20 °C. Hela cells and human fibroblasts for vimentin and cytokeratin staining were seeded on eight‐chamber slides and allowed to attach overnight. Cells were fixed with methanol : acetone 1 : 1 (v/v) for 20 min at −20 °C according to the protocol used for EAC‐SCC cells. These preparations for cytoplasmic antigen staining were additionally permeabilized with PBS containing 0.5% Triton X‐100 and 0.05% NaN3 for 10 min at room temperature.

Following fixation, for cell surface marker staining, preparations were incubated with anti‐CD44‐PE or anti‐CD133‐APC for 1 h at room temperature. For cytoplasmic marker staining, samples were incubated with anti‐pan‐Cytokeratin‐Alexa Fluor^®^ 488 and Vimentin‐PE at room temperature for 1 h, and with anti‐p53‐Alexa Fluor^®^ 488 and anti‐SCCA1/2 (mouse IgG) at 4 °C overnight.

For SCCA1/2 and Oct3/4 antigens, the samples were further stained with goat anti‐mouse IgG (H + L) highly cross‐adsorbed secondary antibody conjugated Alexa Fluor^®^ 555 after the primary antibody reaction. Slides were then mounted with DAPI Fluoromount‐G^®^ (SouthernBiotech, Birmingham, AL, USA) for nuclear staining. Unfixed cytospin preparations were tested with a Vector^®^ Blue Alkaline Phosphatase Substrate kit (Vector Labs, Burlingame, CA, USA) to check for alkaline phosphatase (ALP) activity.

### Immunohistochemistry

Immunohistochemistry was performed on formalin‐fixed, paraffin‐embedded specimens obtained from the EAC tumor samples. Briefly, paraffin sections were cut into 4‐µm‐thick slices with a microtome and mounted on adhesive glass slides (Matsunami Glass Ind., Ltd., Osaka, Japan). After deparaffinization and rehydration, sections were incubated with the primary antibody raised against p53, SCCA1/2, CD44, and fibroblast surface protein (FSP). For the negative control, PBS was substituted for the primary antibody according. After blocking endogenous peroxidase activity with 3% hydrogen peroxide and performing microwave antigen retrieval, the test samples were incubated with biotinylated horse anti‐mouse IgG or biotinylated goat anti‐mouse IgM antibody. Antibody binding was visualized with an avidin‐peroxidase (VECTASTAIN^®^ Elite kit; VectorLabs, Burlingame, CA, USA) and 3,3′‐diaminobenzidine tetrahydrochloride substrate (Dojindo, Kumamoto, Japan). After staining, the slides were washed and dehydrated and coverslips were applied.

### Short tandem repeat analysis

Genomic DNA was extracted from the cell cultures and the tumor samples from the patients using a DNeasy Blood & Tissue Kit (Qiagen, Valencia, CA, USA). The Gene Print PowerPlex 16HS System (Promega, Madison, WI, USA) was used to amplify the extracted DNA. The amplified fragments were detected using an Applied Biosystems 3500xL Genetic Analyzer (Thermo Fisher Scientific).

### P53 DNA sequencing

Genomic DNA was extracted from the EAC‐SCC cell line using a DNeasy Blood & Tissue Kit (Qiagen, Valencia). The extraction of genomic DNA from the fresh‐frozen donor tumor tissues and the patient‐matched skin tissue was conducted using DNeasy Blood and Tissue Kits (Qiagen, Chatsworth, CA, USA). Subsequently, the extracted DNA samples were subjected to whole‐exome sequencing (WES) at the Beijing Genomics Institute (Shenzhen, China). Library preparation was conducted using an Agilent SureSelect All Exon V6 exome capture kit (Agilent Technologies, Santa Clara, CA, USA). The captured libraries were sequenced according to the manufacturer’s protocols using a DNBSEQ‐G400 High‐throughput Rapid Sequencing Set (MGI Tech, Shenzhen, China) with the paired‐end 100‐bp sequence read option. The sequence data were processed via the in‐house pipeline Genomon 2.5.2 (http://genomon.hgc.jp). Briefly, the sequencing reads were aligned to the NCBI Human Reference Genome, Build 37 hg19 with bwa version 0.7.8, using the default parameters (http://bio‐bwa.sourceforge.net/). PCR duplicate reads were removed using Picard (http://www.picard.sourceforge.net). Mutation calling was performed using the EBCall algorithm with the following parameters: (a) mapping quality score ≥ 30; (b) base quality score ≥ 15; (c) both tumor and normal depth ≥ 8; (d) variant reads in tumors ≥ 4; (e) variant allele frequency (VAF) in the tumor samples ≥ 0.05; (f) VAF in the normal samples < 0.1; (g) negative logarithm of the *P*‐value from Fisher’s exact test ≥ 1.3; and (h) negative logarithm of the *P*‐value for EBCall ≥ 5. The mutations in TP53 were visualized using the Integrated Genomic Viewer (IGV, https://software.broadinstitute.org/software/igv/).

### Image capture

Representative culture fields were imaged with the OLYMPUS DP2‐BSW software program using a CCD camera (OLYMPUS DP72) connected to a microscope (OLYMPUS BX51).

## Results

### Preparation of primary tissue culture

**Fig. 1:**
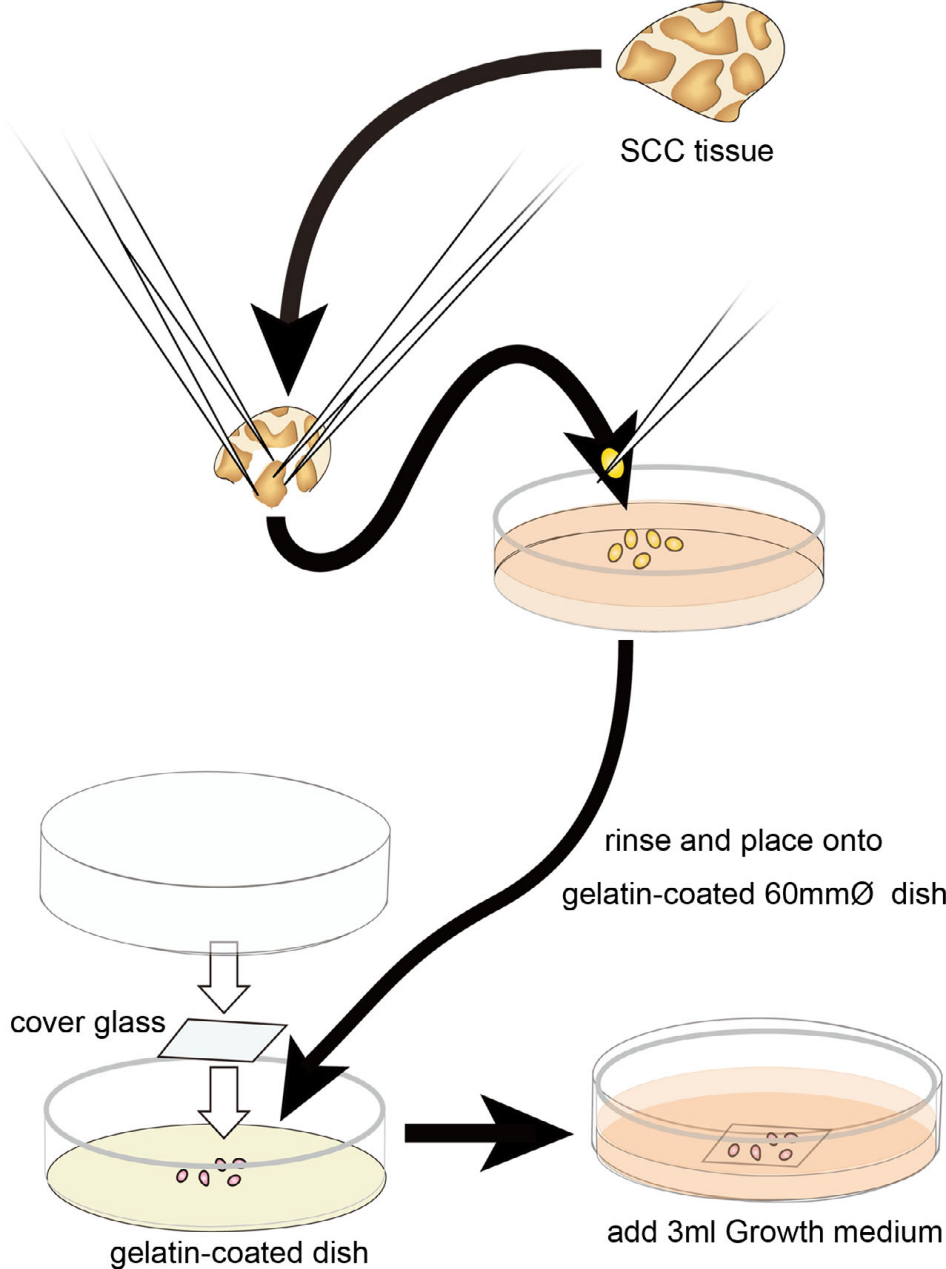
Isolation protocol of SCC‐enriched tissue fragments. Connective tissue, adipose tissue, and blood vessels were removed from the sterilized tumor sample while in the growth medium. The fragments of tumor tissue were placed on a gelatin‐coated 60 mmΦ dish and were pressed‐down with a thick cover glass to keep the tissue from floating. These tissues were cultured in the growth medium until squamous cells and/or cell clusters appeared from the tissue fragments.

Tumor biopsy samples were surgically resected and sterilized by soaking in 99% ethanol for a few seconds to prevent microbial contamination. Thereafter, the tissue was quickly transferred to a sterile container filled with a PBS(−), brought to a clean workbench, and washed with Dulbecco’s PBS (PBS(−): FUJIFILM Wako Pure Chemical Corporation) four times. Connective tissue, adipose tissue, and blood vessels were removed by forceps while in the growth medium. The resulting fragments were placed on a gelatin‐coated 60 mmΦ dish, and pressed‐down with a thick cover glass (Fig. 1). Placing the cover glass on the tissue fragments ensures that they come into contact with the culture surface. As a result, the cells are more reliably extended from the tissue fragment to the culture surface.

### Human‐derived EAC‐SCC cell culture

**Fig. 2:**
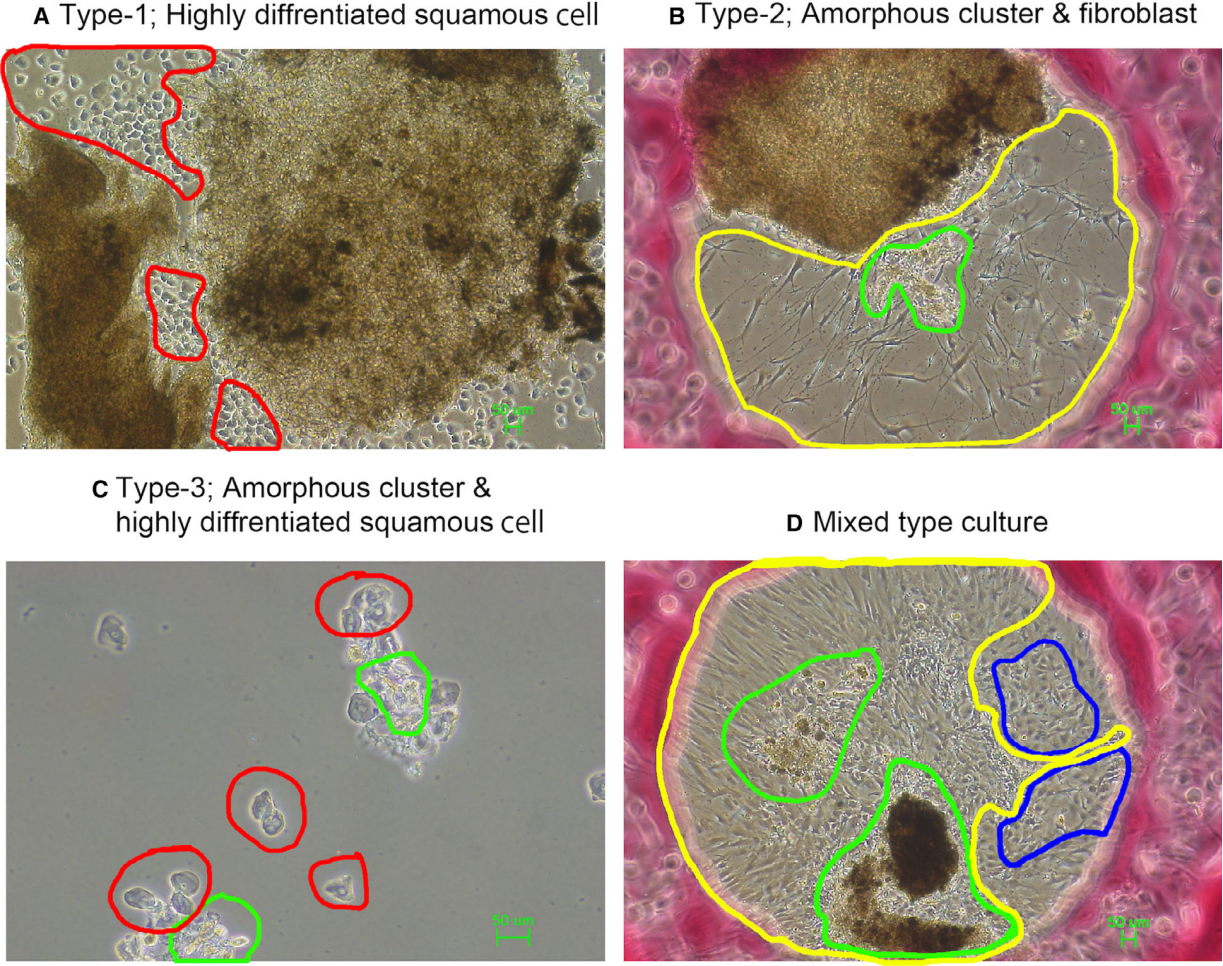
Primary SCC tissue culture. (A) Type‐1 colony: very differentiated squamous carcinoma cells (red circle) leaked from the cancer tissue fragment and spread on the surface of the culture vessel. Scale bar, 50 µm. (B) Type‐2 colony: squamous cell carcinoma cells (green circle) leached out of the cancer tissue fragments as cell clusters, and began to grow on the fibroblasts (yellow circle) instead of the culture surface. Scale bar, 50 µm. (C) Type‐3 colony: highly differentiated squamous cell carcinoma cells (red circle) adhered to the culture vessel surface, and the squamous cell carcinoma clusters (green circle) shown in (B) and (C) adhered and proliferated on those cells. Scale bar, 50 µm. (D) Mixed type culture: epithelial cell‐like squamous cells (blue circle) and fibroblasts (yellow circle) leaked from the same cancer fragments and spread on the surface of the culture vessel. As mentioned in (B), squamous cell carcinoma cell clusters (green circle) were attached and proliferated on fibroblasts. Scale bar, 50 µm.

**Fig. 3:**
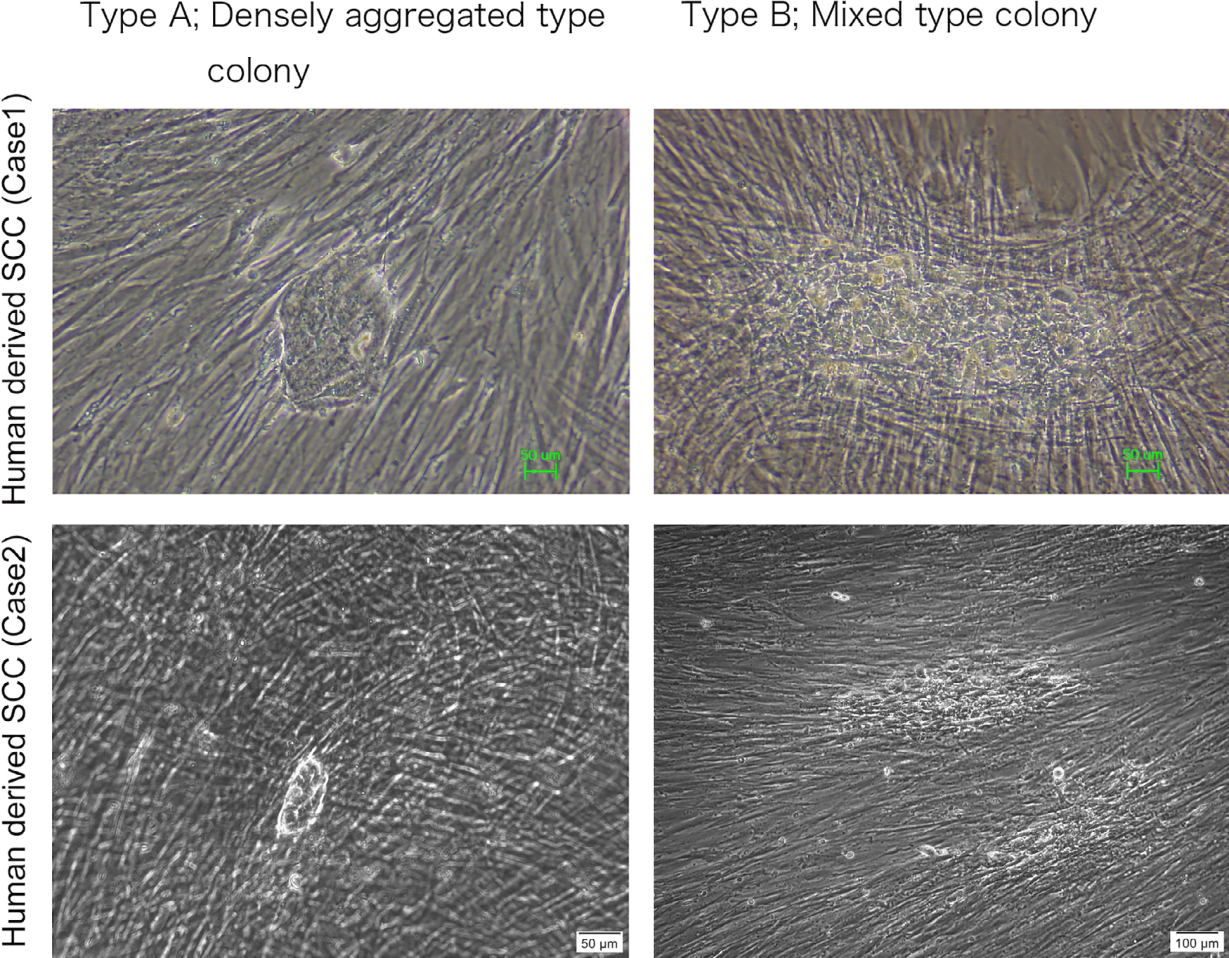
Passaging maintained human‐derived EAC‐SCC colonies in feeder culture. SCC colonies were microscopically picked up from the SCC‐feeder cell culture, dispersed with trypsin, and then spread on feeder cells. During culturing, two distinct types of colonies grew: Type‐A colonies, which were densely packed and which accounted for a large number of the colonies overall, and Type‐B colonies, which formed cell complexes with feeder cells and which were a relatively minor population. Scale bar in both panels of upper row and left panel of lower row, 50 µm. Scale bar in right panel of lower row, 100 µm.

As shown in Fig. 2, several types of cells infiltrated from the cancer tissue fragments and spread on the culture surface to form a colony within several weeks after the start of the culture. In the culture shown in Fig. 2A, highly differentiated squamous epithelium‐like cells (circled in red) were attached to the culture vessel surface (Type‐1 colony). In the culture shown in Fig. 2B, fibroblasts (circled in yellow) exuding from a cancer tissue fragment spread on the surface of the culture vessel. The exuded cancer cells formed an amorphous cell mass (circled in green) (Type‐2 colony) on these fibroblasts. In the culture shown in Fig. 2C, amorphous cancer cell clumps were seen in the cultures on the differentiated squamous epithelial cells instead of the fibroblasts seen in the culture shown in Fig. 2B. In the culture shown in Fig. 2D, multiple types of cells were seeping out from the cancer tissue fragments. In this culture, cells with an epithelial cell‐like morphology (cobblestone‐like uniform sheet) were observed, and this was not seen in other cultures. Colony cells with a unique shape in cultures were picked up with a micropipette tip under a microscope, dispersed by trypsin digestion, and transferred to an MMC‐TIG‐1‐20 feeder culture. Culturing was then continued. As a result, it was possible to continuously culture only the squamous cell carcinoma cells that formed an amorphous cell mass (Fig. 2B–D). Cell cultures from both patients exhibited similar phenotypes. The cells from both patients had a very slow growth rate and a passage rate of once every 4 weeks. This culture was maintained for more than 1 year and was used for subsequent experiments. The differentiated squamous cell carcinoma cells (Fig. 2A,D) and the epithelial cell‐like cancer cells (Fig. 2C) disappeared from the culture during passaging. The fibroblast cells shown in Fig. 2B,C were grown without feeder cells, frozen in liquid N_2_, and used for subsequent experiments. Repeated passaging yielded a cell culture in which two types of homogeneous colonies were reliably and reproducibly formed (Fig. 3). Type A colonies are densely packed and occupy a large proportion of all colonies. Type B colonies have indistinct boundaries characterized by cell–cell complexes with TIG‐1‐20 fibroblast feeder cells.

### Cancer cell markers of tissue specimens

**Fig. 4:**
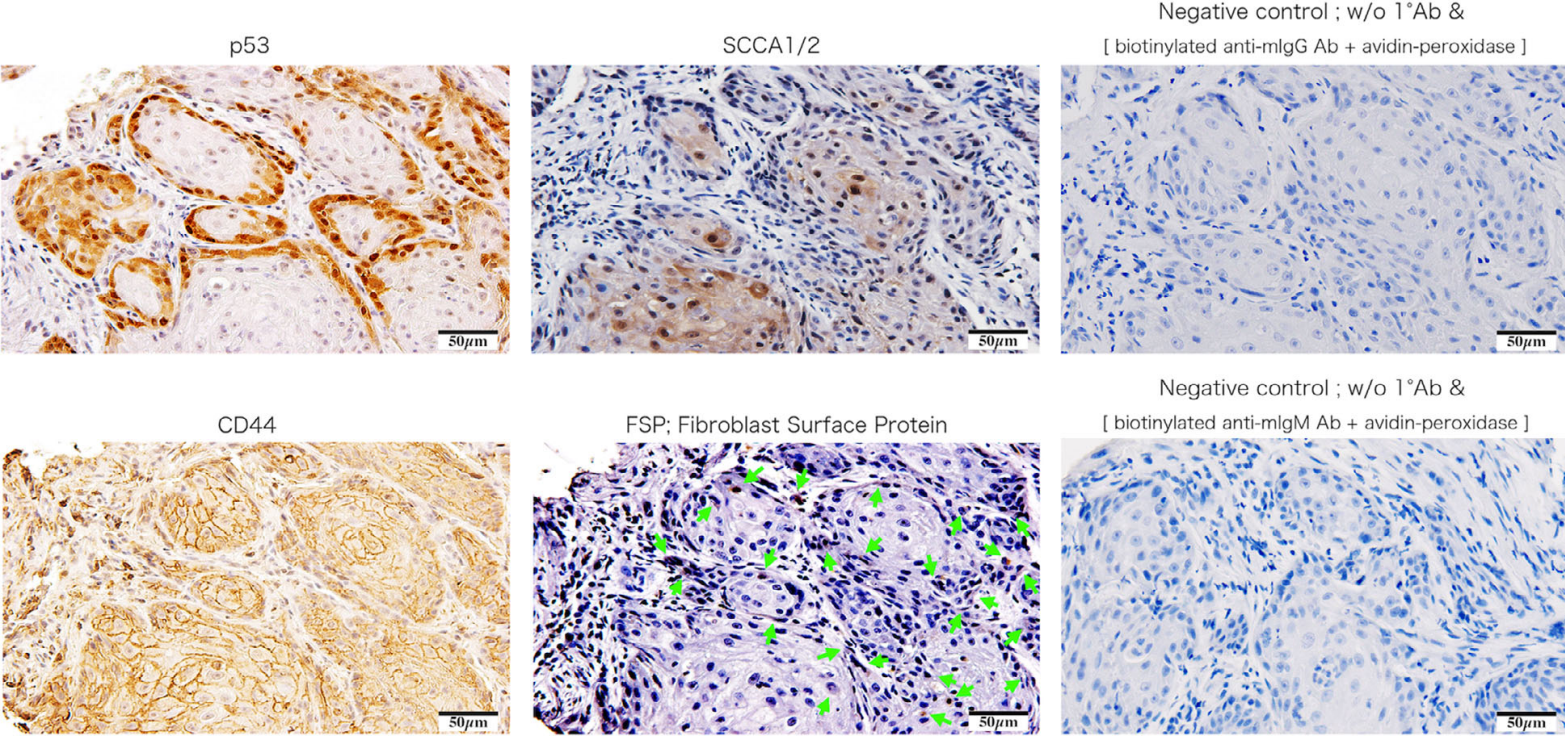
Immunohistochemical staining of squamous cell carcinoma tissue of the external ear canal. Histological specimens from the original donor were stained with p53, SCCA1/2, CD44, and FSP. p53‐positive cells, and SCCA1/2‐ and CD44‐positive cells were localized to the outer shell of the tumor lobe and tumor core, respectively. Fibroblasts were mainly distributed around the tumor lobe. Scale bar, 50 µm.

Histological specimens from the original donor were examined to investigate the distribution of cells positive for p53, SCCA1/2 [20], CD44, and FSP [21]. p53‐positive cells were mainly localized to the outer shell of the tumor lobe. In contrast, SCCA1/2‐ and CD44‐positive cells were preferentially located in the tumor core. Fibroblasts were mainly distributed around the tumor lobe (Fig. 4).

Somatic mutations of TP53 are the most frequent driver mutations in head and neck squamous cell carcinoma [22]. Therefore, to explore whether the donor EAC‐SCC tumor tissues harbored somatic mutations of TP53, and whether the established cell culture retained identical alterations, we performed WES on genomic DNA derived from the cell culture and the donor tumor tissue (from case 2) and used a patient‐matched skin tissue sample as a noncancerous control. WES and the subsequent bioinformatics analysis revealed that both the cell line and the donor tumor tissues had a nonsynonymous mutation in exon 7 of TP53 (TP53G245S), which is reportedly a hotspot mutation in TP53 (variant allele frequency: 8.3% and 49.3% in the donor tumor tissue and the cell culture, respectively) [23]. Thus, the established cell culture had retained the important genetic alteration of the donor EAC‐SCC (Fig. S1).

### Cancer cell markers in human‐derived SCC cells

**Fig. 5:**
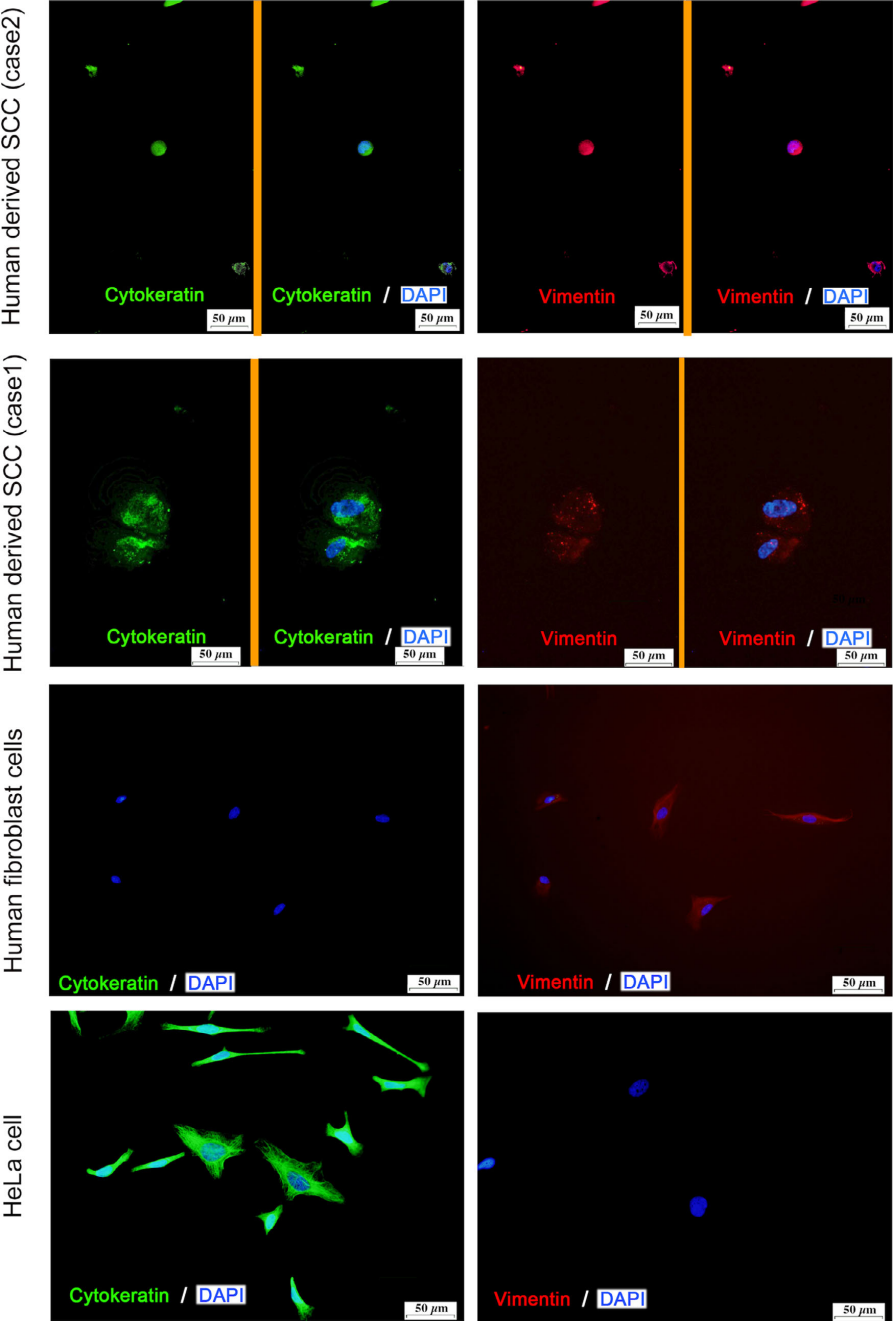
Fluorescence immunostaining with anti‐cytokeratin‐Alexa 488 (green) and anti‐vimentin‐PE (red). EAC‐SCC cells were positive for both anti‐pan‐Cytokeratin‐Alexa 488 (green) and anti‐Vimentin‐PE (red). Cytokeratin and vimentin were located in the cytoplasm but not in the nuclei. Human fibroblasts were positive for anti‐Vimentin‐PE (red), but negative for anti‐pan‐Cytokeratin‐Alexa 488 (green). Hela cells were positive for anti‐pan‐Cytokeratin‐Alexa 488 (green) but not anti‐Vimentin‐PE (red). Scale bar, 50 µm.

**Fig. 6:**
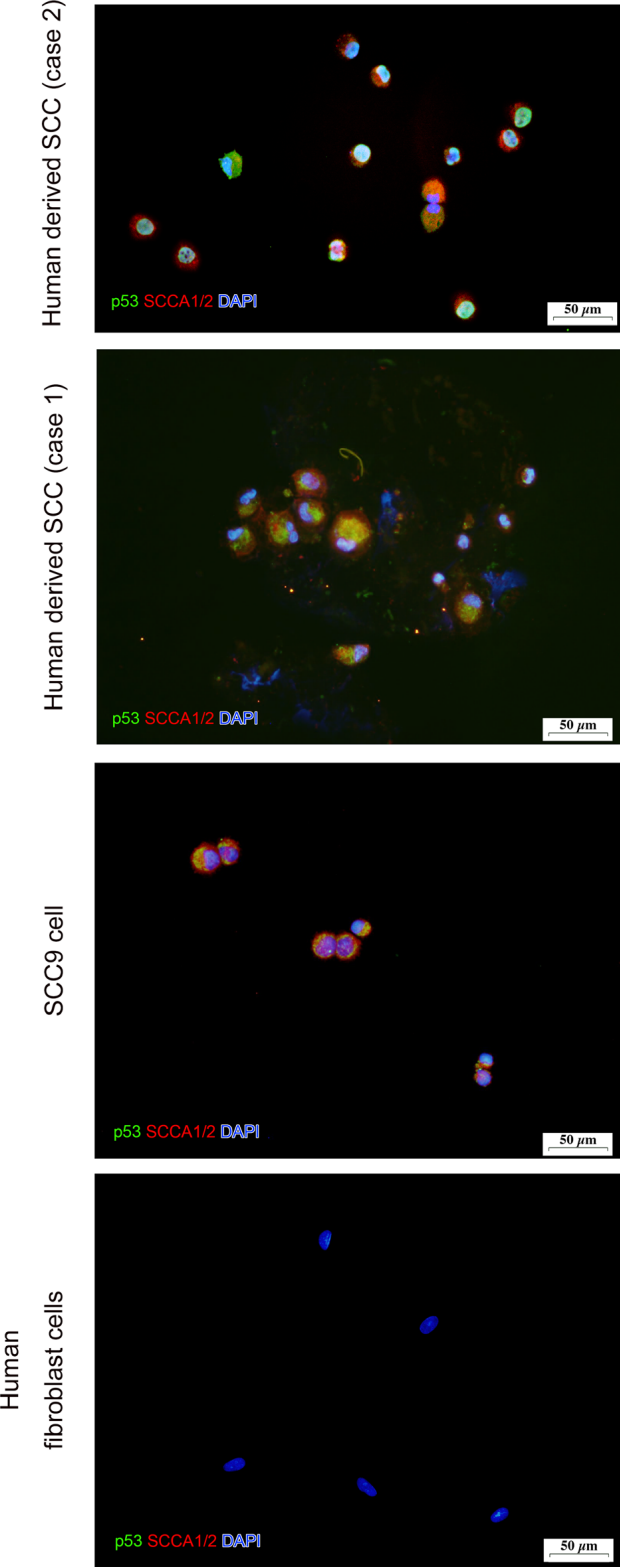
Fluorescence immunostaining with cancer‐specific antibodies. Cells were prepared on slides using Cytospin 4^®^ (Thermo Fisher Scientific). The cells were double‐stained with anti‐p53‐Alexa 488 (green) and anti‐SCCA1/2 + Alexa Fluor^®^ 555 anti‐mouse IgG (red). Our cells were double‐positive for p53 and SCC. On the other hand, human fibroblast cells were negative for both anti‐p53‐Alexa 488 (green) and anti‐SCCA1/2 + Alexa Fluor^®^ 555 anti‐mouse IgG (red). Scale bar, 50 µm.

**Fig. 7:**
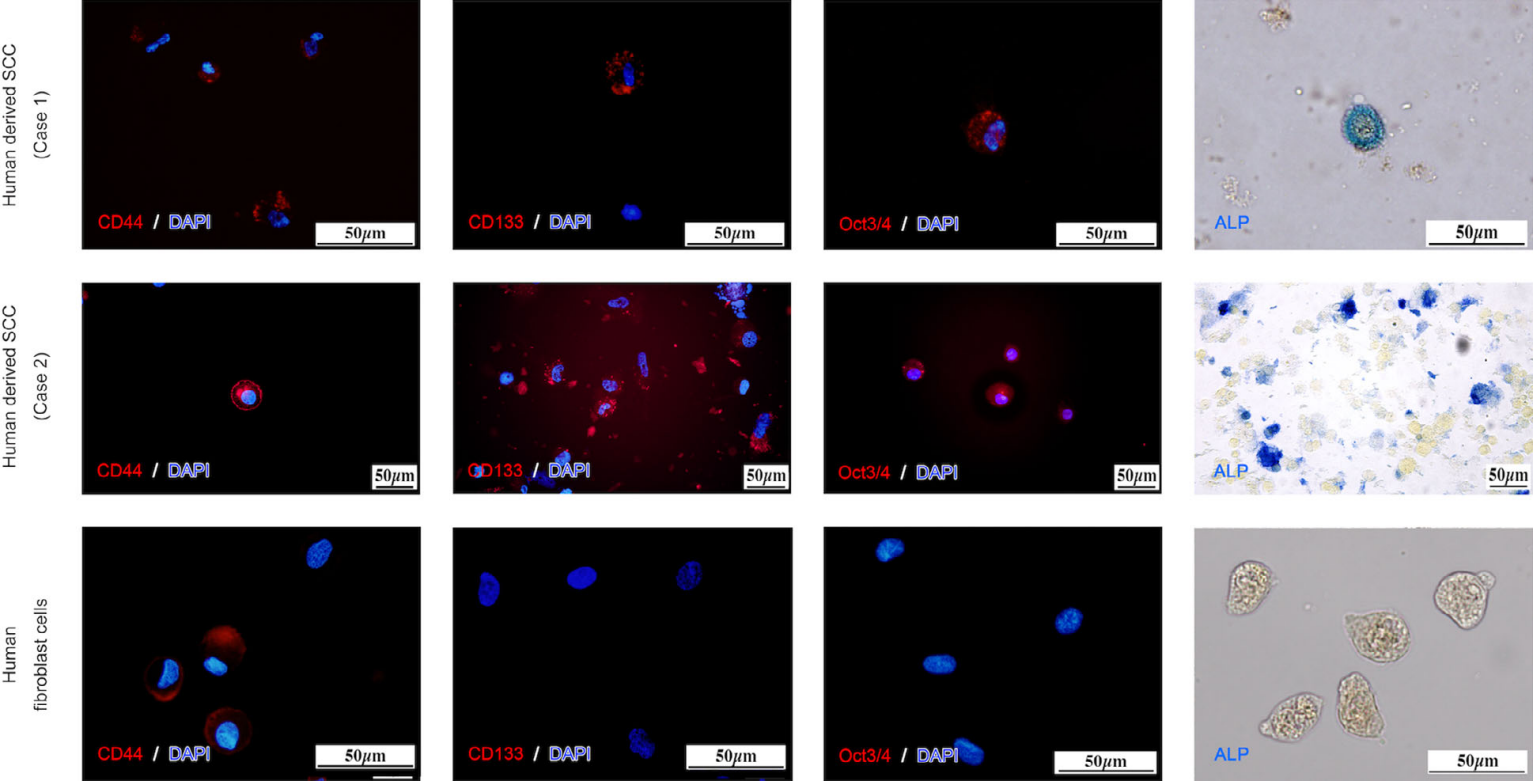
Examination of cancer stem cell markers in EAC‐SCC cells. Cells were prepared on slides using Cytospin 4^®^ (Thermo Fisher Scientific). EAC‐SCC cells were tested with anti‐CD44‐PE, anti‐CD133‐APC, and anti‐Oct‐3 / 4 + Alexa Fluor^®^ 555 anti‐mouse IgG. DAPI (blue) was used to identify the location of cell nuclei for immunofluorescence staining. Fibroblasts were positive for CD44 and were not stained with CD133 or Oct3/4. In addition, ALP activity was observed in our EAC‐SCC cells but not in fibroblasts. Scale bar, 50 µm.

Double immunostaining experiments for cytokeratin–vimentin and p53–SCCA1/2 were performed to verify that these cells were SCC cells derived from the donor tumor tissue. Immunocyto‐staining revealed that the cells were double‐positive for both cytokeratin and vimentin showed, suggesting the possibility of malignancy [24, 25, 26] (Figs 5 and 6). In order to examine the stemness of these cells, colonies of these cells were excised under a microscope and dispersed by trypsin/EDTA treatment, and the presence of cancer stem cell markers (CD44 [14, 16, 17], CD133 [14, 15, 16, 17], Oct3/4 [14, 18], ALP [16]) was examined in these free cells. The EAC‐SCC cells were found to be positive for these cancer cell markers (Fig. 7).

### Macrophages disperse EAC‐SCC colonies

External auditory canal cancer has an extremely poor prognosis, often involving metastatic infiltration from the primary focus into the surrounding tissue and accompanying inflammation. Therefore, to test how EAC‐SCC colonies behave in the presence of inflammatory cells, the EAC‐SCC cell–feeder culture was monitored for changes after adding human macrophages.

**Fig. 8:**
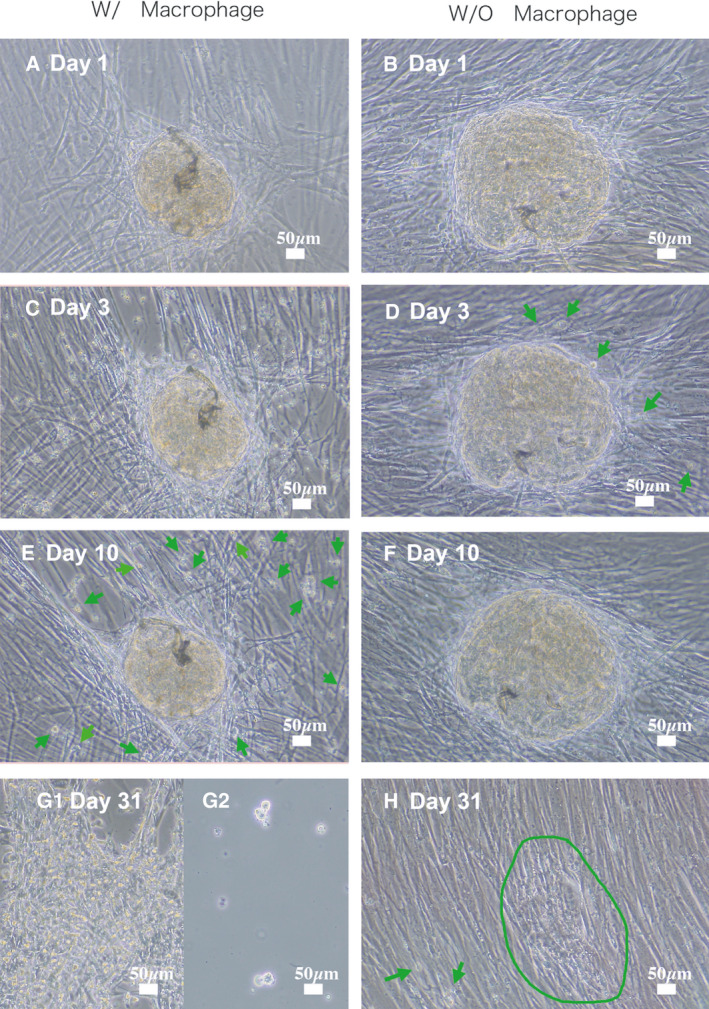
Transformation of explosively spreading colonies of human‐derived SCC cells. (A, B) Peripheral blood mononuclear cells (PBMCs) were plated on day 1 over colonies of our cells. Free cells were discarded when the culture medium was replaced every three days. The cultures were maintained for one month. (C) Macrophage had bounded to many cells, including EAC‐SCC cell–fibroblast complexes on day 3. (D) Some cancer cells migrated from the colony. (E, F) By day 10 after culture initiation, lymphoid cells disappeared from the culture, and only macrophages remained as cells derived from PBMCs. (G, H) EAC‐SCC cells co‐cultured with macrophages did not form exclusive colonies consisting only of EAC‐SCC cells and formed steric complexes with some of the healthier feeder cells. EAC‐SCC cells without macrophage kept to form the colony. Green arrows show migrated cancer cells. (A–F), (G‐1), (H): ×100. (G‐2): ×200 Scale bar, 50 µm.

First, colonies were scraped from the EAC‐SCC cell–feeder culture by micropipette tip under microscope, then seeded on a feeder culture. Once the adherence of the new colonies to the medium and growth was confirmed, fresh macrophages were layered on the culture (Fig. 8A: EAC‐SCC with macrophage and 8B: EAC‐SCC cell without macrophage). On day 3, macrophages had bound to many cells, including EAC‐SCC cell–fibroblast complexes, and started to penetrate inside the clusters (Fig. 8C,D). On day 10, EAC‐SCC cells had started to disperse from colonies, migrating over the surrounding feeder cells (Fig. 8E). While this trend was similar to the cells’ normal proliferative behavior (Figs 3 and 8F,H), one key difference was that they did not form exclusive colonies consisting only of EAC‐SCC cells. However, they did form steric complexes with some of the healthier feeder cells (Fig. 8G‐1). Fibroblast‐bound cells ranged from 5 to 20 µm in size. Trypan blue dye exclusion tests were performed to confirm that these cells were not macrophages: While smaller (< 5 µm) cells were dyed blue, none of the > 20‐µm cells were dyed blue (Fig. 8G‐2). This suggests that the cells of least 20 µm in size within the complexes were EAC‐SCC cells.

In normal cultures without macrophages, some cells broke away from the EAC‐SCC colonies (Fig. 8D), forming new ones and proliferating in a distant area (Fig. 8H). This phenomenon had the appearance of normal proliferation, but the cells did not disperse and proliferate as they did in the EAC‐SCC cell–macrophage cultures.

### Short tandem repeat analysis

The cell colonies from case 1 were too small to extract sufficient DNA for analysis. The doubling time of the cells derived from case 1 was slower than that of the cells derived from case 2. Additionally, the cells derived from case 2 formed larger colonies than those of case 1. We were thus able to extract sufficient DNA from the cells cultured from case 2. The short tandem repeat (STR) profile of the cell culture did not match that of the host tumor sample exactly, but the STR similarity between the cell culture and host tumor sample was 0.96, which was sufficiently high that the STR profile of the cell culture could be considered the same as that of the host tumor sample. We, therefore, consider these cells to be an identical cell strain. In addition, we confirmed that there were no other identical cell lines in the Japanese Collection of Research Bioresources (JCRB; Table S1).

## Discussion

In the present study, we successfully cultured human EAC‐SCC cells. These cell cultures have several unique characteristics (Table 3):

1 Colony formation with a steric structure characterized by cell–cell complexes with MMC‐treated TIG‐1‐20 fibroblasts
2 Very slow growth
3 Double positivity for SCCA 1/2 and p53 antigens, hallmarks of SCC
4 Positivity for several cancer stem cell markers (CD44, CD133, Oct3/4, ALP)
5 Double positivity for cytokeratin and vimentin

**Table 3:** Characteristics of human derived EAC‐SCC cells.

Cell features	
Colony formation	Steric structure with fibroblasts Small colony senses to grow further
Growth rate	Very slow
Immunohistochemistry	
SCC markers	p53, SCC
Cancer stem cell markers	CD44, CD133, Oct3/4, ALP
Diagnosis and prognosis prediction	Double positivity for cytokeratin and vimentin

This evidence supports the conclusion that the established cell culture did originate from SCC cells in the EAC, and that it possesses many of the characteristics of cancer stem cells. One of the stem cell markers, ALP, is a membrane‐bound enzyme. Four isoenzymes have been found in various tissues: intestinal ALP, placental ALP, germ‐cell ALP, and tissue‐nonspecific ALP. High levels of ALP have been reported to be a tumor marker for various cancers [27]. Many types of pluripotent stem cells, as well as iPS cells, are known to have ALP activity [28, 29, 30]. Expression of cancer stem cell markers, including CD44, CD133, Oct3/4, and ALP activity, may be one reason the growth of our cell cultures was suppressed. Furthermore, in addition to cytokeratin, our cell cultures were positive for vimentin, a marker of a poor prognosis in head and neck cancer [24, 25, 26].

Derived from an EAC‐SCC tumor, our cells formed steric colonies on the fibroblast feeder layer culture, but never on the surface of the culture vessel. One possible reason for the difficulty in creating sustainable *in vitro* cultures of EAC carcinomas is their difficulty in adhering to the culture vessel; successful cultures seem to require a scaffold of fibroblast feeder cells.

Since our cells were positive for cancer stem cell markers [14, 15, 16, 17], one might assume they are preferentially found in the interior of tumor lobes (a hypoxic environment) *in vivo* [31]. Unusually, immunostaining found that most of the p53‐positive cells were located on the exterior of the tumor lobe. Given its role in cell division, the p53 expression level within tumor masses (where cell cycle arrest is maintained by the hypoxic environment) is low. However, it seems reasonable to assume that the process would restart, and that the cells would start to proliferate, once brought into *in vitro* conditions with sufficient oxygen, at which point they would be p53‐positive. These histological findings strongly suggest that our cells originate from cancer cells in the tumor interior, a hypothesized cancer stem cell niche [31].

*In vitro*, the EAC‐SCC cells formed steric structure colonies on the feeder layer culture. We performed immunostaining of donor tumor tissue with an FSP antibody to further investigate this phenomenon. Fibroblasts were primarily located at the periphery of the tumor tissue, although a small number were present in the interior, suggesting that fibroblasts possess cadherins or other cell adhesion molecules with affinity for our cells, which mediate the latter’s maintenance and growth.

In the clinical setting, vimentin positivity is considered to be indicative of a poor prognosis [24, 25, 26]. SCC cells are known to express this molecule and exhibit enhanced migration potential as they transform into mesenchymal stem cells, a process known as the epithelial–mesenchymal transition [32]. Our cell cultures exhibit both of these properties.

PDC has been reported as a negative prognostic factor, which is established in several types of cancer [10, 11, 12]. EAC‐SCC cells form an amorphous cluster with feeder layer cells and/or with the same cells. Also, when proliferating, cells migrating from the cluster create satellites around this cluster and increase. This growth pattern appears to be a potential homolog of PDC seen in the invasive front of tumor budding *in vivo*. In addition, when co‐cultured with human macrophages derived from peripheral blood for long periods, the EAC‐SCC cell–fibroblast complexes slowly change to facilitate break‐away and dispersal. We presume that macrophages probably play some role in the human‐derived SCC colony dissociation. Thus, we propose our cell cultures as a potential ‘key cell’ in research to explore how changes in the tissue microenvironment caused by macrophage invasion, a trigger for inflammation, affect how SCC metastasizes and spreads.

## Conclusion

In this study, we established and maintained cell cultures from human EAC‐SCC, and unlike normal SCC cells, these formed amorphous cell clusters only on feeder cells, not on the culture vessel surface. Our cell cultures possess many of the characteristics of cancer stem cells. These cell cultures could thus be used in future research on EAC‐SCC.

## Conflict of interest

The authors report no personal financial support for this article and no conflicts of interest regarding any of the materials or devices described in this article.

## Author contributions

NK involved in conceptualization. YS, AI, and MO performed the formal analysis. NK and TN contributed to funding acquisition. HO and TN performed the investigation, validation, and project administration. YS, AI, MO, AF, and TH involved in resources. NK supervised the article. YS, AI, and NK wrote original draft. YS, AI, NK, SM, AF, KK, KS, and RU reviewed and edited the manuscript.

## Supporting information


**Fig. S1.** P53 sequencing analysis in human‐derived SCC. Somatic mutation in exon 7 of TP53 (TP53G245S), visualized using the Integrated Genomic Viewer (IGV). The sequencing coverage, reads, and altered bases in skin tissue (top), donor tumor tissues (middle), and the cell line (bottom) are shown.

**Table S1.** Short tandem repeat (STR) profiles.

## Data Availability

The authors confirm that the data supporting the findings of this study are available within the article and its supplementary materials.
