# Supplementary figures and images for: Establishment and characterization of a primary cell culture derived from external auditory canal squamous cell carcinoma
Source: FEBS Open Bio. 2021 Jun 29;11(8):2211–24. doi: 10.1002/2211-5463.13225 (PMC8329851; doi:10.1002/2211-5463.13225)

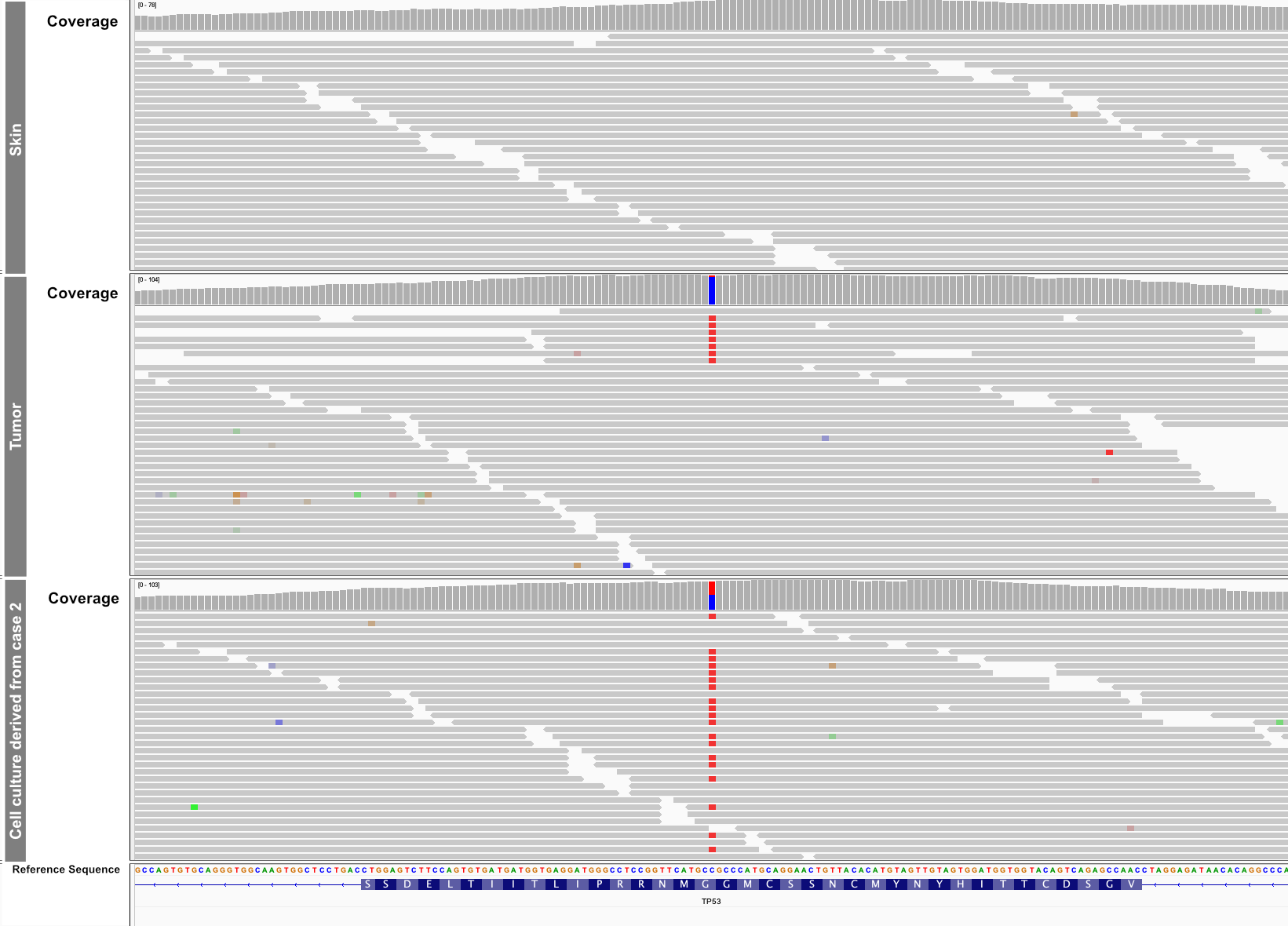

Supplement: Supplementary file 1 — Fig. S1. P53 sequencing analysis in human‐derived SCC. Somatic mutation in exon 7 of TP53 (TP53G245S), visualized using the Integrated Genomic Viewer (IGV). The sequencing coverage, reads, and altered bases in skin tissue (top), donor tumor tissues (middle), and the cell line (bottom) are shown. [file FEB4-11-2211-s002.tiff]
