# Supplementary material for: Establishment and characterization of a primary cell culture derived from external auditory canal squamous cell carcinoma
Source: FEBS Open Bio. 2021 Jun 29;11(8):2211–24. doi: 10.1002/2211-5463.13225 (PMC8329851; doi:10.1002/2211-5463.13225)
Supplement: Supplementary file 2 — Table S1. Short tandem repeat (STR) profiles. [file FEB4-11-2211-s001.docx]

**Supplemental table 1. Short tandem repeat (STR) profiles**

Short tandem repeat values of cell culture and tumor tissue for fifteen gene loci and amelogenin (X and Y chromosomes). Evaluation value between cell culture and host tumor tissue was 0.96, which was high enough that STR profile of cell culture are the same as those of host tumor sample.
